# Unlocking potential: innovative “private-non-profit” partnership for empowering children with disabilities in resource-limited settings in Nepal

**DOI:** 10.3389/fpubh.2025.1438992

**Published:** 2025-02-19

**Authors:** Bibek Banskota, Rajan Bhusal, Prakash Kumar Yadav, Jagdish Lal Baidya, Ashok Kumar Banskota

**Affiliations:** ^1^Hospital and Rehabilitation Centre for Disabled Children (HRDC), Banepa, Nepal; ^2^B&B Hospital, Lalitpur, Nepal

**Keywords:** physical disabilities, pediatric healthcare, resource-limited settings, private-nonprofit partnership, community-based rehabilitation, Nepal

## Abstract

**Background:**

Physical disabilities affect approximately 240 million children globally, with limited access to comprehensive care in resource-constrained settings. In Nepal, an estimated 2% of children under 16 experience physical disabilities, facing significant barriers to healthcare access, education, and social integration. Traditional healthcare models often struggle to provide affordable, accessible, and sustainable care for these children.

**Objectives:**

To evaluate the effectiveness and sustainability of an innovative private-nonprofit partnership model between the Hospital and Rehabilitation Centre for Disabled Children (HRDC) and B&B Hospital in Nepal, designed to provide comprehensive care for children with physical disabilities in resource-limited settings.

**Methods:**

The study analyzes a 40-year experience implementing a unique healthcare delivery model combining HRDC’s non-profit expertise with B&B Hospital’s private sector resources. The model integrates four key components: identification through mobile camps and community outreach, comprehensive medical treatment, rehabilitation services, and social reintegration programs.

**Results:**

The partnership achieved a 62% reduction in treatment costs compared to private healthcare institutions. Over 40 years, HRDC has performed more than 55,000 surgeries, benefiting over 116,000 children surgically. The program has distributed 100,000+ assistive devices, raised disability awareness among 1.5 million+ people, and trained over 700 primary rehabilitation therapists. The model’s community-based approach has enabled coverage of all 77 districts in Nepal through rotating mobile clinics.

**Conclusion:**

The HRDC-B&B partnership demonstrates that private-nonprofit collaboration can effectively address healthcare barriers for children with physical disabilities in resource-limited settings. The model’s success in combining cost efficiency, quality care, and community integration provides a replicable framework for similar interventions in other developing countries. Key factors for success include diverse funding sources, strong community engagement, and integrated service delivery under one roof.

## 1 Introduction

### 1.1 Importance of addressing physical disabilities in children

Globally, the World Health Organization (WHO) estimates that over 240 million children live with disabilities, with approximately 10% of them experiencing physical disabilities. These disabilities often arise from congenital conditions, injuries, infections, or malnutrition, disproportionately affecting children in low-and middle-income countries (LMICs) (1). In Nepal, a population-based study revealed that approximately 2% of children under 16 years’ old experience some form of physical disability, representing a significant public health burden (2). Early identification and intervention are critical as untreated physical disabilities can lead to secondary complications, including deformities, chronic pain, and impaired development (3). Furthermore, the socio-economic impacts are substantial; children with disabilities are less likely to attend school, with UNICEF reporting that 85% of children with disabilities in developing countries remain out of school (4).

### 1.2 Global challenges and barriers faced by children with physical disabilities

Children with physical disabilities face a multitude of challenges worldwide. According to UNICEF, children with disabilities are nearly four times more likely to experience violence and neglect compared to their peers without disabilities (5). Globally, only 5–15% of children needing assistive devices or mobility aids have access to them due to cost, availability, and lack of technical expertise (6). In Nepal, systemic barriers include limited access to specialized healthcare facilities, particularly in rural areas where 83% of the population resides. A study conducted by the National Federation of the Disabled Nepal (NFDN) found that 61% of children with disabilities lacked access to rehabilitation services, and only 20% of children with severe physical disabilities attended school due to mobility challenges and stigma.^1^

### 1.3 The need for effective health intervention models

Traditional public healthcare systems in resource-limited settings often struggle to meet the complex needs of children with physical disabilities. For example, Nepal’s government allocates only 1.2% of its GDP to healthcare (7), with limited provisions for specialized pediatric disability services. To address these gaps, innovative health intervention models are essential. Evidence suggests that partnerships between private and non-profit sectors can deliver sustainable and high-impact solutions. For instance, the Hospital and Rehabilitation Centre for Disabled Children (HRDC) in Nepal (8), a non-profit organization, provides holistic care to over 25,000 children annually through collaborative efforts with private donors and technical partners.

#### 1.3.1 Children with physical disabilities in Nepal

With a population of 29,164,578 and a Human Development Index (HDI) of 0.602, Nepal exhibits the lowest HDI among South Asian countries (9, 10). An estimated 2.2% of the total population in Nepal has some form of disability. This comprises 2.5% of the male and 2.0% of the female population; 36.75% of total disability is attributed to physical disabilities, and an estimated 8.7% have multiple disabilities (11). According to the United Nations, an estimated 60,000 to 180,000 children aged 5 to 14 in Nepal live with disabilities, predominantly physical disabilities (12).

Common categories of physical disabilities in Nepal include congenital, neuromuscular, trauma, infections, burns, metabolic, or tumor-related conditions. Addressing the comprehensive needs of these children requires a continuum of care encompassing identification, surgical interventions, and ongoing support across preventive, promotive, curative, and rehabilitative dimensions (13). Regrettably, despite the critical nature of these needs, children with physical disabilities often face societal neglect, hindering their access to essential services such as healthcare (14), education, and other opportunities. In the Nepalese context, these individuals encounter challenges stemming from the limited availability, accessibility, and affordability of dedicated services for disability treatment and rehabilitation (3).

In Nepal, there is a paucity of specialized treatment options for children with physical disabilities. Although existing private and government institutions address some aspects of care, a significant gap persists in delivering truly comprehensive treatment models for children with disabilities, especially those who come from socioeconomically disadvantaged backgrounds (12).

Private services, mostly catering to urban populations, present a challenge due to their higher costs and limited accessibility. The financial constraints exacerbate healthcare disparities for vulnerable groups, further widening the gap in access to comprehensive treatments for children with physical disabilities in Nepal. Despite significant strides in recent years, the lives of children with physical disabilities in Nepal remain marked by a complex web of challenges (3). In a country where the rural population predominates, children with disabilities born into low-resource families face the barriers of care availability, affordability, and often acceptability, the latter related to the various prevalent stigmas that negatively influence care-seeking behavior (15).

Thus, a program modeled to make care “available” by taking the hospital to the child’s doorsteps, “affordable” by virtue of a non-profit charitable model, and “acceptable” by educating the families and communities to seek care for their disabled as opposed to conforming to stigmas and rituals, is the need of the hour if we are to tackle this enormous problem.

The purpose of this model and article is to propose and evaluate an innovative “Private–Non-profit” partnership approach to address the unmet needs of children with physical disabilities in resource-limited settings, with a focus on Nepal. The model seeks to bridge gaps in healthcare access, provide holistic rehabilitation, and ensure social reintegration by leveraging the strengths of private sector resources and non-profit expertise. The article aims to contribute to the global discourse on healthcare interventions by offering a scalable, cost-efficient, and sustainable framework. By demonstrating the model’s effectiveness, the study aspires to inform policy, empower communities, and enhance the overall quality of life for children with disabilities, fostering inclusion and equity in global health.

## 2 Literature review

Children with physical disabilities face significant challenges globally due to social, economic, and infrastructural barriers, which adversely affect their quality of life and limit their potential. Social stigma and discrimination are persistent challenges, with children often being excluded from community activities and social interactions. According to a UNICEF study (1), children with disabilities are nearly four times more likely to experience violence compared to their non-disabled peers. This heightened vulnerability often results in severe psychological effects and social marginalization. Globally, at least 15% of the population—amounting to over one billion individuals—live with some form of disability, whether congenital or acquired later in life. Among them, nearly 240 million are children, highlighting the significant proportion of young individuals facing such challenges (1). Cultural beliefs in some regions, particularly in low-and middle-income countries (LMICs), perpetuate negative attitudes, where disabilities are often misunderstood or associated with superstitions (16).

Economically, families with children with disabilities often face additional financial burdens, including costs for specialized healthcare, assistive devices, and transportation. According to the World Bank (17), households with disabled members are 20% more likely to experience poverty due to these added expenses. Infrastructural barriers further exacerbate these challenges. For instance, inaccessible transportation systems and lack of ramps or elevators in schools and public buildings hinder mobility, limiting access to education and healthcare services (18).

Healthcare remains another critical area of concern. Children with disabilities often struggle to access essential medical services due to a lack of trained healthcare professionals and limited availability of assistive technologies. Assistive technology encompasses physical devices (e.g., wheelchairs, prosthetics, hearing aids) and digital solutions (e.g., speech recognition, time management tools) that improve cognitive, sensory, and physical functioning, enabling inclusion and participation in education, work, and daily life. While over 2.5 billion people currently need assistive products—a number expected to reach 3.5 billion by 2050—access remains highly inequitable, with only 3% of people in low-income countries accessing the products they need compared to 90% in high-income countries. Barriers include high costs, limited awareness, inadequate policies, and a workforce gap (6).

Globally, 240 million children live with disabilities, facing significant disadvantages in education, health, and well-being compared to their peers. They are less likely to attend school, access health services, or experience inclusive care and more likely to face malnutrition, discrimination, and abuse. Barriers like stigma, lack of inclusive services, and physical and communication obstacles perpetuate these gaps, leaving many excluded from opportunities to reach their potential (19).

There are very few models that provide comprehensive treatment to children with physical disabilities, as most existing approaches focus on isolated aspects of care, such as medical or surgical interventions while neglecting rehabilitation, psychosocial support, and societal reintegration. This fragmented approach highlights significant gaps in achieving holistic outcomes. Further research is needed to develop integrated frameworks that address the diverse needs of these children, combining medical, therapeutic, educational, and social interventions. Additionally, innovation is essential in creating community-based support systems and leveraging technology-driven solutions, such as assistive devices and telemedicine, to enhance accessibility and the overall effectiveness of care.

### 2.1 Prevalent models for non-profit hospitals

Non-profit hospitals worldwide utilize various funding models to sustain their operations, each presenting unique advantages and challenges. Here’s an in-depth look at these models.

#### 2.1.1 Individual donations

Relying on individual contributions, this model fosters community engagement and a sense of ownership among donors. The flexibility of these funds allows hospitals to address immediate needs without stringent restrictions (20). However, the unpredictability of donation levels can lead to financial instability, and maintaining a steady flow of contributions requires ongoing, resource-intensive fundraising efforts.

#### 2.1.2 Family foundations

Support from family foundations often comes in the form of substantial grants, enabling hospitals to undertake large projects or establish endowments. These foundations may offer multi-year funding, aiding in strategic planning and long-term initiatives (21). Nonetheless, such funds may come with specific conditions, limiting the hospital’s flexibility in resource allocation. Additionally, over-reliance on a single foundation poses risks if the foundation’s priorities shift.

#### 2.1.3 Government support

Government funding provides a stable financial base, ensuring operational continuity and aligning hospital services with public health objectives. However, this model subjects hospitals to bureaucratic constraints and potential red tape, which can impede swift decision-making. Moreover, economic downturns or policy changes can lead to budget cuts, affecting the sustainability of services (22).

#### 2.1.4 NGO support

Partnerships with non-governmental organizations (NGOs) can enhance service delivery to target populations, bringing in additional resources and international expertise. However, differing organizational goals may lead to conflicts, and NGO funding is often project-based and time-limited, raising concerns about long-term sustainability (23).

#### 2.1.5 Mixed model: Narayana Health

Narayana Health in India exemplifies a mixed funding model, combining elements of for-profit efficiency with philanthropic principles. This approach allows revenue from paying patients to subsidize care for those who cannot afford it, enhancing accessibility. High patient volumes contribute to economies of scale, reducing per-unit costs and enabling affordable services. The organization also adopts cost-saving technologies and processes to maintain quality while reducing expenses (24). However, replicating this model in regions with different economic conditions can be challenging, and maintaining high-quality care amid large patient volumes requires rigorous management (25).

Each funding model offers unique benefits and faces specific challenges. Non-profit hospitals often adopt a combination of these models to balance financial sustainability with their mission to provide accessible healthcare.

To the best of our knowledge, there is no existing public-private partnership model specifically designed to address the needs of children with physical disabilities. Based on the success and sustainability of our own model, which has been effectively running for over 40 years, we believe it has the potential to be replicated in other low-resource settings. While our partnership was facilitated by the unique circumstance of having our founder involved on both sides, this approach could just as effectively be implemented by two or more like-minded individuals or institutions sharing a common vision. In our case, that vision is the treatment and rehabilitation of socioeconomically disadvantaged children with musculoskeletal disabilities.

## 3 Materials and methods

The study employs a comprehensive case study approach to analyze the 40-year successful implementation of the HRDC-B&B Hospital partnership model (1985–2024) in Nepal, focusing on its sustainability and effectiveness in delivering healthcare to children with physical disabilities. This longitudinal analysis is structured around three primary domains. The first domain, partnership structure analysis, examines the formal and informal arrangements between HRDC (a non-profit) and B&B Hospital (a private institution). It evaluates governance mechanisms, resource-sharing agreements, operational protocols, financial frameworks, cost-sharing models, and stakeholder roles and interactions. The second domain, service delivery model analysis, explores patient care pathways from identification to rehabilitation, assesses the hub-and-spoke model connecting the central facility with satellite clinics, and evaluates mobile camp strategies and community outreach programs. It also documents referral systems and cross-institutional collaboration. The third domain, impact assessment, combines quantitative and qualitative analyses. Quantitative analysis covers treatment data over 40 years, including the number of surgeries performed (>55,000), total beneficiaries (>116,000 children), distribution of assistive devices (>100,000), and geographic coverage (77 districts). Qualitative analysis includes case studies of successful interventions, stakeholder interviews, and documentation of community impact and social integration outcomes.

The methodology relies on diverse data collection methods, including reviewing 40 years of institutional records, analyzing financial and operational documents, conducting semi-structured stakeholder interviews, collecting patient testimonials and success stories, and documenting organizational processes and protocols. To ensure reliability and validity, the study incorporates triangulation of multiple data sources and addresses limitations, such as historical data gaps, through cross-verification with stakeholders and supporting documentation. This comprehensive approach aims to provide a detailed understanding of the partnership model’s success in delivering sustainable healthcare services over four decades, with a particular emphasis on identifying replicable elements for similar global settings.

## 4 Public-private partnerships model

### 4.1 Introduction to HRDC

**Figure 1:**
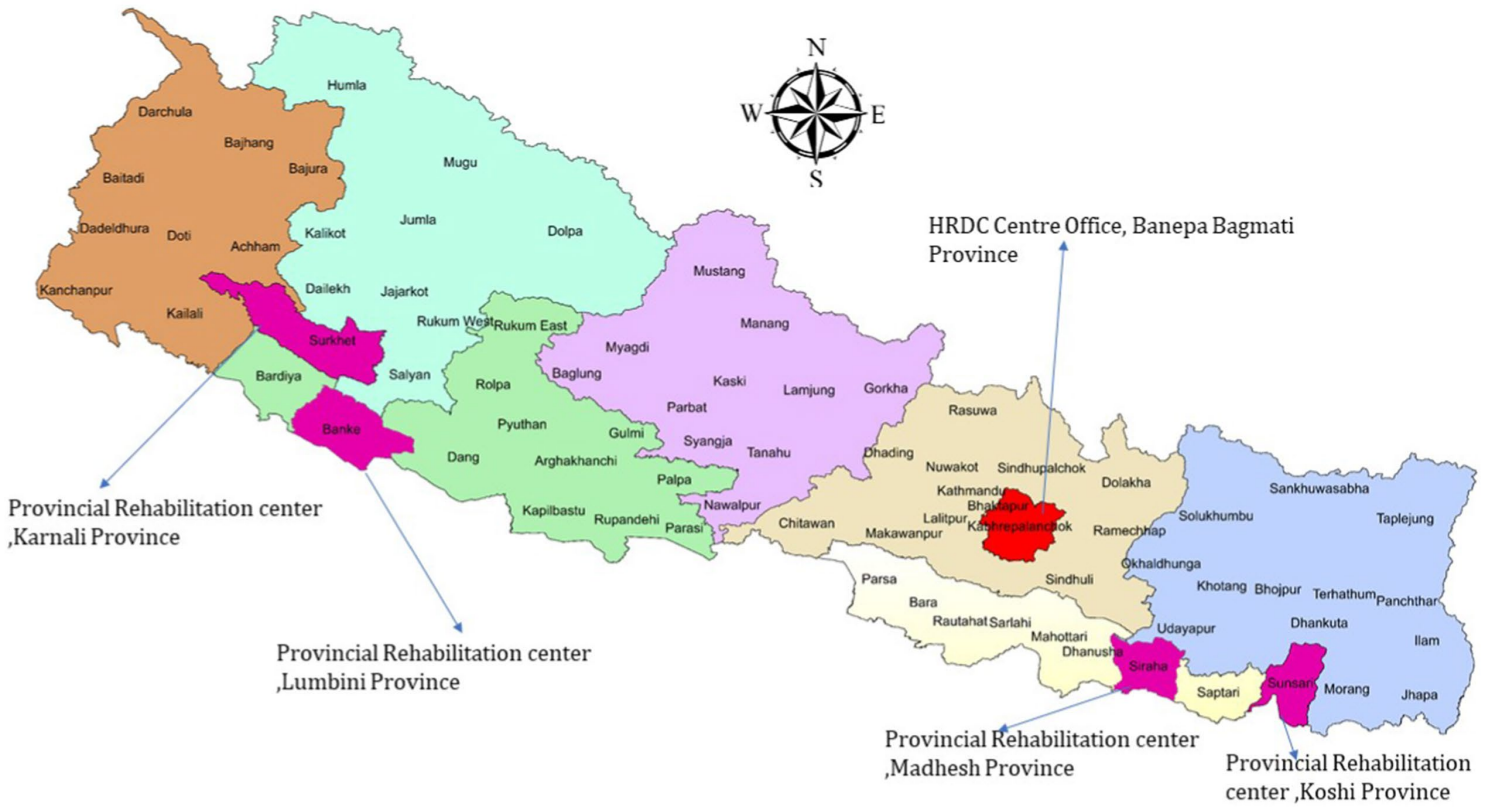
HRDC center and satellite clinics (The 77 districts of Nepal are covered once every 3 years by HRDC’s extensive mobile clinic network).

The Hospital and Rehabilitation Centre for Disabled Children (HRDC) is a specialized tertiary care facility with 100 beds dedicated to pediatric orthopedic conditions. A non-profit organization founded in 1985 by Dr. Ashok K Banskota, HRDC is committed to offering comprehensive care for children with physical disabilities in Nepal.^2^ Approximately 25,000 consultations and 2,500 major reconstructive pediatric orthopedic surgeries are performed annually. The HRDC’s target population is children with physical disabilities who come from socioeconomically disadvantaged families. Our robust community-based rehabilitation (CBR) approach ensures that our mobile consultation and follow-up clinics scout target children from the nooks and corners of Nepal and bring them for treatment at our mother center, a state-of-the-art hospital facility near the capital, Kathmandu. The HRDC operates four satellite clinics, two each in the east and west of Nepal, which orchestrate our field program that includes organizing mobile clinics, measurement and fabrication of orthotic and prosthetic devices, conducting awareness-education-advocacy campaigns for disability rights, and networking and liaison with local stakeholders to ensure wider awareness of our services. The clinics also provide essential follow-up care and physiotherapy services. This decentralized model allows many of our beneficiaries to access comprehensive services at our satellites, significantly enhancing convenience and accessibility for those in need and mitigating treatment dropouts (Figure 1).

### 4.2 Need for the establishment of the HRDC in Nepal

The establishment of the Hospital and Rehabilitation Centre for Disabled Children (HRDC) in Nepal in 1985 AD was driven by a crucial need to address the diverse requirements of disabled children in low-income settings. At that time, the founder, who had received orthopedic training in the United States, returned to Nepal and observed a significant lack of targeted facilities for children with physical disabilities. In the early stages, the founder noted that existing hospitals lacked orthopedic-trained surgeons, and while some foreign doctors were present, the absence of well-equipped facilities hindered specialized care. Consequently, many children with complex deformities were left untreated and often found on the streets. Existing services were primarily focused on general treatments rather than the specific needs of children with physical disabilities. Not only were children deprived of the necessary healthcare and rehabilitation services, but the societal challenges surrounding disabilities were also prevalent. Social stigmas, taboos, and broader social problems added significant complexity to the issue. Disabled children faced not only a dearth of medical attention but also encountered discrimination and societal prejudices that hindered their overall well-being. This situation was observed during the inception of HRDC in 1985 AD and remains pertinent in the current context.

The establishment of HRDC sought to address the medical necessity of providing comprehensive treatment and the societal imperative of tackling deeply ingrained stigmas and prejudices. By contributing to the reduction of societal stigma associated with disabilities, HRDC aimed to create a more inclusive and compassionate environment. By implementing educational programs, awareness campaigns, and support initiatives, HRDC helped shift public attitudes, emphasizing the capabilities and rights of individuals with disabilities rather than their limitations. This effort not only promoted a better understanding of disabilities but also raised empathy and compassion, encouraging communities to embrace inclusivity and respect for diversity. Through these initiatives, HRDC contributed to dismantling deeply ingrained stereotypes and stigmas, creating a more supportive and welcoming environment for everyone.

The institution recognized that empowering disabled children goes beyond medical interventions—it involves equipping them with the skills and support necessary for leading fulfilling lives.

Fast forward to 2024, HRDC remains Nepal’s only pediatric orthopedic hospital providing comprehensive treatment and rehabilitation for children with physical disabilities. The significance of HRDC lies in its ability to address the multifaceted challenges faced by disabled children in Nepal, encompassing healthcare, education, and social inclusion.

### 4.3 Approaches of the HRDC

The HRDC working model consists of two main components: one within the hospital and the other in the community. Adopting a holistic approach, the HRDC focuses on addressing physical disabilities in children through four key areas: identification, treatment, rehabilitation, and social reintegration. These areas work in a continuous cycle, ensuring a comprehensive and sustainable solution that improves the quality of life for children with musculoskeletal disabilities. The strategies implemented aim not only to provide immediate medical care but also to foster a supportive environment for long-term recovery and societal integration. Below is a detailed description of the activities within each of these areas:

*Identification:*

- Mobile Camps: Conducted in the community to actively identify children with physical disabilities.
- Referral Mechanisms: Collaboration with organizations and institutions for effective referrals.
- Awareness Campaigns: Community-based campaigns to promote early identification and reduce stigma.
- Home Visits and Follow-Up: CBR workers visit homes to identify new children who have a physical disability and track the progress of those under treatment or rehabilitation.
- Network of Central Hospital and Satellite Clinics: Directing children to suitable services for evaluation and treatment.
- Collaboration with previously treated patients: Encouraging community referrals through education and providing examples of successfully treated patients.
- Teleconsultation services that link the mother Centre with the satellite and mobile clinics ensure prompt assessment and decision-making, thus avoiding multiple visits and attrition.

*Treatment:*

- Comprehensive care “under one roof” covers all aspects of pediatric orthopedic musculoskeletal disability.
- Services include diagnostics, surgery, physiotherapy, and an in-house orthotic prosthetic lab manufacturing low-cost assistive devices/ artificial limbs.

*Rehabilitation:*

- Physical rehabilitation:
  - Physiotherapy services to improve levels of function
  - Occupational therapy to improve activities of daily living (ADL)
  - Assistive devices/ artificial limbs to improve ambulation and social participation
- Social rehabilitation:
  - Child protection policy ensures that treated children return to conducive environments at home.
    - A dedicated child protection officer educates families visiting the hospital and, where necessary, intervenes and liaises with local authorities to ensure that children do not become victims of exploitation or stigma.
  - An in-house school is used for the ongoing education of admitted children

*Social Reintegration:*

**Figure 2:**
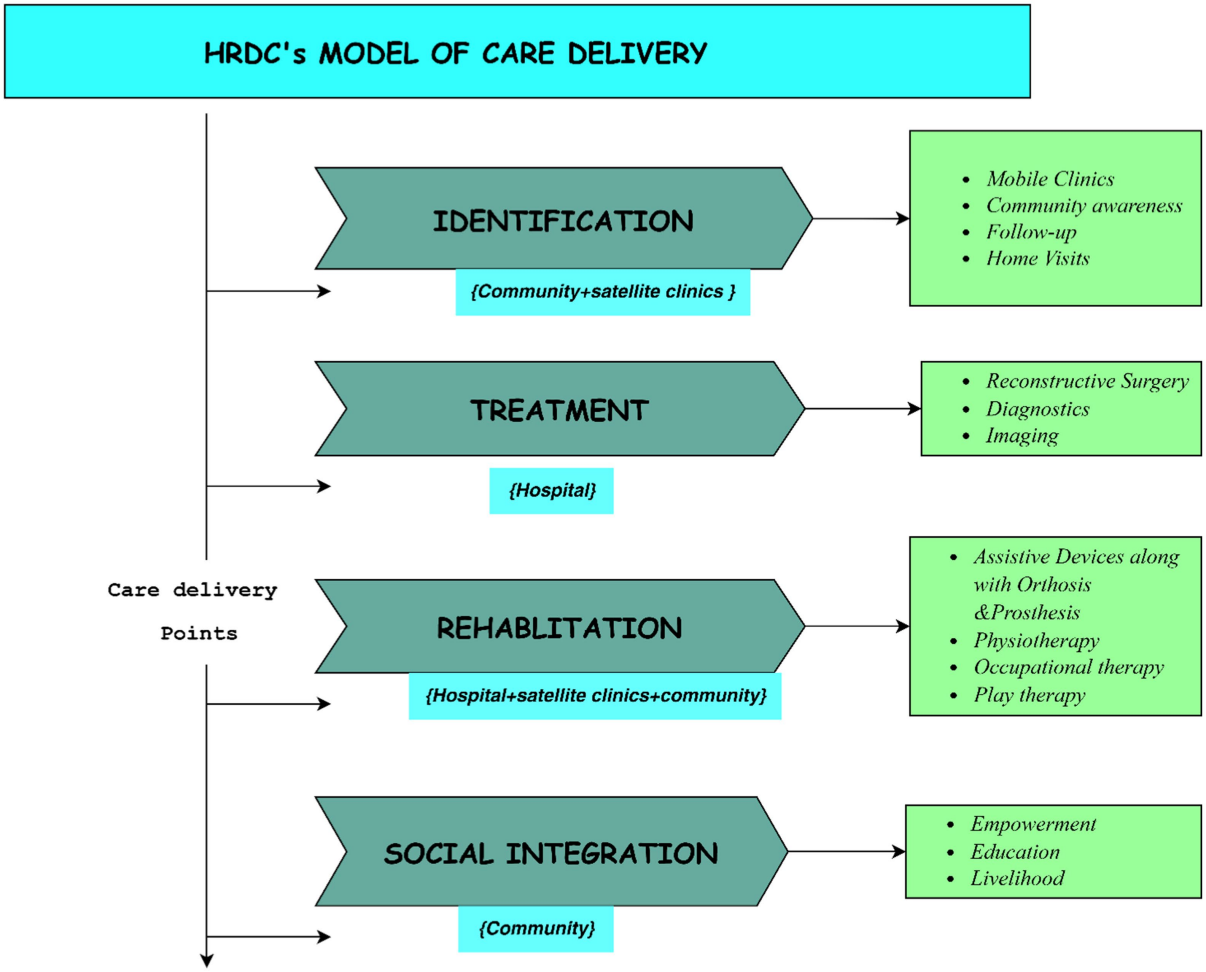
HRDC’s model of care delivery for children with physical disabilities.

**Figure 3:**
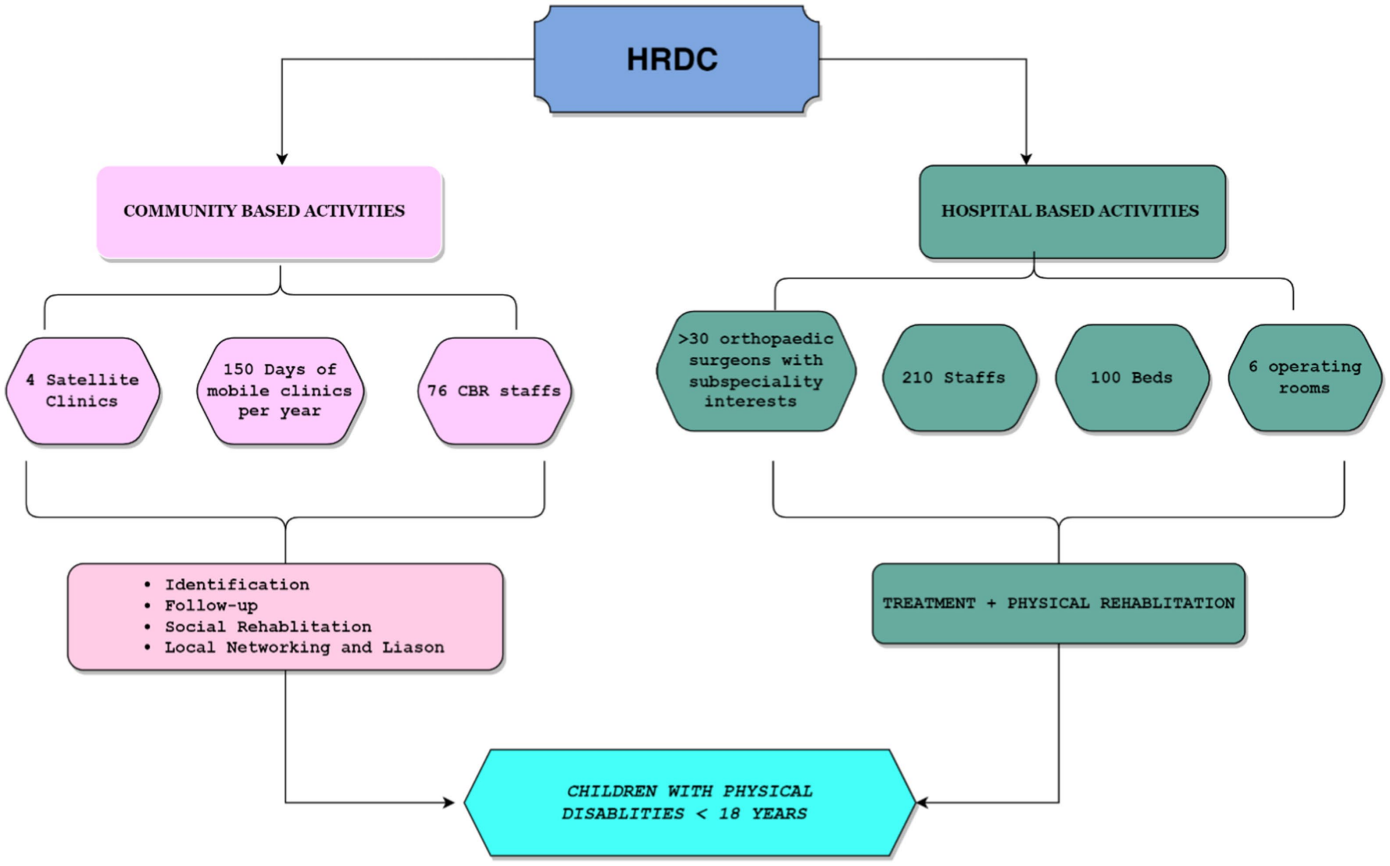
Community and hospital-based activities provided by the HRDC.

- Emphasis on Community-Based Rehabilitation (CBR) for seamless integration into communities.
- Collaboration with local government bodies, disability organizations, schools, and guardians.
- Focus on mainstream education, community participation, and livelihood opportunities.
- Continuous collaboration with CBR workers for ongoing support and sustained efforts in social reintegration (see Figures 2, 3).

### 4.4 Governance and funding at HRDC

The HRDC, a non-profit organization with over 200 staff, operates under a participatory management model guided by an executive and hospital management team. Governance is overseen by a diverse Board of Directors, ensuring strategic direction and adherence to the organization’s mission. Funding, crucial for HRDC’s humanitarian and development work, comes from international donations, grants, government contributions at various levels, and personal donations. This diversified funding approach enhances financial stability and flexibility, allowing HRDC to respond to evolving needs and effectively impact its mission.

### 4.5 Private institution: introduction to for-profit hospital

The for-profit (B&B Hospital) is a 300-bed private hospital renowned for its orthopedic services. With a team of 120 doctors and over 800 staff members, the hospital sees over 70,000 new patients across all disciplines annually, admits over 9,000 inpatients, and conducts approximately 14,000 surgeries.^3^ Regarding musculoskeletal disorders, B&B hospital has an advanced imaging Centre with modern CT-scan, MRI, DEXA scan, and Scannogram facilities. HRDC utilizes this at a significantly subsidized cost. B&B hospitals, modern operative theatres, intensive care units, and pediatric departments support HRDC patients who require complex interventions. For example, a child requiring complex spinal deformity correction has all advanced imaging done at B&B and may be operated at B&B hospital if the above support is necessary in the pre-or postoperative period. Any other medical, surgical, pathology, etc. input available at B&B is accessible to patients from HRDC.

### 4.6 Collaboration of non-profit and for profit

The collaboration between HRDC (a non-profit hospital) and B&B (a private organization) has proven to be a symbiotic partnership, significantly benefiting HRDC and ensuring its sustainability. The multifaceted support provided by B&B encompasses skilled personnel, high-end diagnostic services, and underprivileged patient referrals from B&B to HRDC, creating robust support for HRDC’s operations.

**Figure 4:**
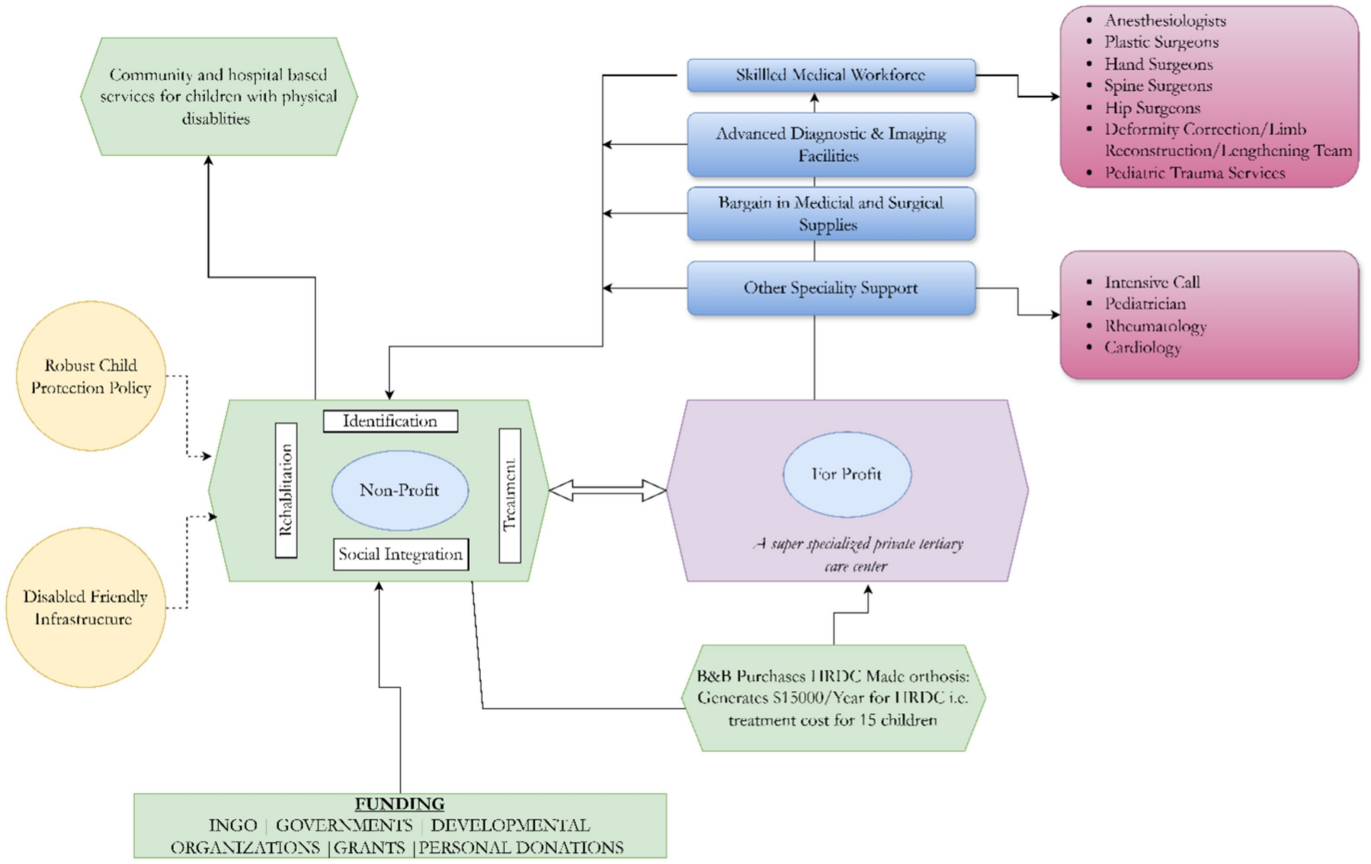
Collaboration model between non-profit and for-profit.

- *Skilled Personnel*: The Orthopedic department at B&B hospital comprises a 30-strong team of surgeons subspecialized in trauma, spine, hip-pelvis, hand reconstruction, and limb-lengthening. While B&B Hospital provides a platform for private practice and livelihood, all team members contribute time to HRDC for a meager remuneration. HRDC operates 5 days a week, with surgeries fixed for Tuesdays and Wednesdays. Surgeons also provide outpatient consultations on the other 3 days. Each surgeon rotates, working an average of 2 days per week at HRDC and the remaining days at B&B. Thus, a wealth of specialized orthopedic services are made available at HRDC without a corresponding increase in cost that would occur should HRDC have to pay hefty salaries for these skilled personnel. The total cost incurred in such remuneration is less than 5% of the total budget of the HRDC, thus reserving vital resources for patient care.
- *Diagnostic Services*: B&B’s provision of high-end diagnostic services, including MRI and CT scans at minimal costs, is a game-changer for HRDC. By sparing HRDC the expenses associated with acquiring, maintaining, and managing such equipment, B&B allows the non-profit organization to allocate its scarce resources elsewhere. This cost-saving measure directly contributes to HRDC’s financial sustainability, enabling it to redirect funds toward patient care, staff welfare, and facility improvements. Also, radiologists at B&B hospital contribute time to HRDC for a small remuneration, thus saving more resources.
- *Cost-Effective Access to Medical Equipment*: B&B’s role in acquiring expensive medical equipment further underscores the collaborative synergy. Through established business arrangements, B&B ensures that HRDC can access state-of-the-art medical equipment at a subsidized market price. This arrangement applies to medicines (pharmacy) and other commodities used at both hospitals. This bargain represents an effort towards sustainability, enabling HRDC to stay abreast of technological advancements in healthcare without compromising its financial stability.
- *Patient Referrals*: Beyond technical and diagnostic support, B&B is crucial in extending HRDC’s reach to patients who cannot afford private healthcare services. By referring non-affording families who have children with musculoskeletal disorders to HRDC from B&B, B&B contributes to the non-profit organization’s mission of providing inclusive and affordable healthcare. This broadens HRDC’s impact on the community and aligns with its commitment to serving those with limited financial means (see Figure 4).

### 4.7 Mutual benefits of the collaboration (sustainability factors within partnership)

- Private Sector Benefits

- High-quality private institution (B&B Hospital) provides excellent earning potential.
- Strong academic and research opportunities through the HRDC
- Enhanced professional networking
- Prestigious social standing in the medical community
- Retention of skilled professionals due to attractive compensation and benefits

- Non-profit Service Benefits

- Spiritual fulfillment through charitable service
- Community goodwill and social recognition
- Opportunity to serve underprivileged populations
- Access to and experience with diverse cases from across the country.
- Professional satisfaction through impactful work
- Enhanced sense of community ownership and social responsibility

- Symbiotic Relationship

- A win-win situation for both institutions
- Private practice earnings enable voluntary /minimum remuneration service at non-profits.
- The cross-referral system benefits both institutions
- Shared expertise and resources optimize operations
- Professional growth opportunities in both settings

### 4.8 Sustainability of the model

#### 4.8.1 Organizational sustainability

Leadership Stability and Vision:

- The founding chairperson and executive director provide robust leadership.
- Long-term commitment to sustainability through their vision for the organization.
  Human Resource Development:
- HRDC is dedicated to training employees.
- Scholarships are arranged for employees at different levels, including surgeons and nurses.
- Focus on developing expertise through different tiers of training.
  Infrastructure and Property:
- Own buildings and land, including a central office and a provincial hospital in the east.
- Liaison with the government for office premises in various locations, ensuring a stable physical presence.
  Nationwide Network and Collaboration:
- A robust network of Community-Based Rehabilitation (CBR) workers throughout the country.
- Regular training for CBR workers and collaboration with different NGOs and INGOs.
- Development of referral mechanisms and sustainable networks.
  Employee Retention and Expertise:
- Low staff turnover, with employees serving for durations ranging from 1 to 36 years.
- Mixed expertise of both young and experienced professionals.
  Medical Reporting System:
- A robust medical reporting and recording system is in place, ensuring accurate and timely documentation.

#### 4.8.2 Community sustainability

Affordability, Acceptability, and Accessibility:

- Affordability: providing services at no/low cost.
- Acceptability: Tailoring services to diverse cultural needs throughout the country.
- Accessibility: Providing convenient services on their doorsteps through the mobile camps, home visits, and follow-ups through community-based rehabilitation.
  Goodwill and Unique Care:
- Leveraging goodwill for community trust.
- Offering a distinctive, patient-centered care approach.
  Preventive Services:
- Conducting awareness and stigma reduction programs.
- Focusing on early detection and lifestyle education.
  Government Engagement:
- Collaborating with different government levels for comprehensive healthcare strategies.
  Promotive, Treatment, and Rehabilitative Services:
- Promotive: Initiating wellness and health promotion campaigns.
- Treatment: Providing medical interventions tailored to individual needs.
- Rehabilitative: Supporting recovery and functional improvement.

#### 4.8.3 Financial sustainability

HRDC operates as a cost-efficient, non-profit healthcare model, significantly reducing treatment expenses compared to private institutions. Private healthcare costs (Cₚ) include base treatment costs (B), profit margins (20–25%), doctor charges (20–25%), and asset depreciation (10–12%), resulting in total costs of approximately 2.63B. In contrast, HRDC eliminates profit margins, minimizes doctor charges, and relies on donated infrastructure and equipment, reducing costs to just the base treatment cost (Cₕ = B). This leads to a 62% cost reduction, allowing HRDC to operate at only 38% of the cost of private institutions.

The remaining 38% of costs are covered through donations, grants from local and international organizations, voluntary contributions, and optional patient payments. HRDC ensures no child is denied care due to financial constraints. This model motivates donors by maximizing their impact, enabling scalable initiatives to underserved areas, and aligning with their goals of providing sustainable, high-impact healthcare.

By offering affordable and accessible care, HRDC addresses children’s immediate healthcare needs and fosters donor trust and community resilience, proving that compassion-driven healthcare can scale effectively and sustainably. Detailed cost breakdowns are provided in Supplementary material.

##### 4.8.3.1 Why donors are motivated?

The 62% cost reduction achieved at HRDC is a significant incentive for donors because it allows them to:

1. Maximize Impact: With HRDC’s efficiency, donors can help treat more children for the same funding amount compared to private institutions.
2. Scale Initiatives: Reduced costs make extending services to more remote and underserved communities feasible.
3. Align with Goals: Donors are driven by the desire to see their contributions create widespread change, and HRDC’s model embodies their mission of sustainable, high-impact healthcare.

##### 4.8.3.2 Affordable and accessible care for all

The HRDC model demonstrates that:

- 62% cost savings make providing high-quality care at a fraction of the private sector’s cost possible.
- The remaining 38% of funding is sourced from generous donors, ensuring no financial burden is placed on patients.
- This approach aligns with the vision of inclusive healthcare, making treatment affordable, accessible, and impactful for children with physical disabilities in Nepal.

By leveraging its cost-efficient model, HRDC addresses children’s immediate needs and strengthens donor trust and community resilience, proving that compassion-driven healthcare can scale effectively and sustainably.

## 5 Impact and achievements

Over 40 years, HRDC has profoundly impacted the lives of children with disabilities. More than 116,000 children have benefitted from surgical interventions, with over 55,000 surgeries performed. The organization has also played a crucial role in raising disability awareness, reaching over 1.5 million people and significantly changing societal attitudes towards disabilities. In addition, HRDC has fabricated and distributed more than 100,000 assistive devices, providing essential tools to improve the quality of life for children with physical disabilities.

The center has been instrumental in clubfoot treatment, having successfully treated over 18,000 cases, making it one of the largest clubfoot centers in the world. HRDC’s dedication to knowledge sharing is evident in the publication of 150+ articles in national and international journals, contributing valuable research to the global medical community. Every year, HRDC performs around 2,000 surgeries for children, with an average of three surgeries needed for complete treatment, demonstrating their commitment to comprehensive care.

Beyond clinical work, HRDC is dedicated to training and developing professionals. The center has trained over 700 primary rehabilitation therapists and annually educates 300 orthopedic surgeons through workshops and conferences nationwide. Moreover, HRDC has advocated for disability management, designing and implementing disability management courses in more than 60 districts out of 77 nationwide, ensuring that disability care and support are integrated into local healthcare systems across the country.

HRDC has been honored with several prestigious global awards for its outstanding achievements. These include the World of Children Award in 2011, recognizing its exceptional contributions to improving the lives of children.^4^ In 2014, HRDC was awarded the Stars Foundation Award from the UK, highlighting its impactful work in disability and healthcare.^5^ The organization was further acknowledged with the World of Children Alumni Award in 2016, reinforcing its commitment to children’s health and welfare (see text footnote 4). Most recently, in 2022, HRDC received the TKS Gold Medal, a testament to its continued excellence and dedication to transforming the lives of children with disabilities. These accolades reflect HRDC’s enduring influence and leadership in the global healthcare community.

### 5.1 Testimonials from beneficiaries

A Young Boy’s Story*: “HRDC corrected my feet and provided both education and employment. The treatment gave me a new life, and I now go by the name Amal, which means ‘hope.’”*

A Teenager’s Transformation*: “After undergoing burn contracture surgeries, the transformation was miraculous. I can now attend school, make friends, and even dream of a future in politics.”*

A Girl’s Journey to Independence*: "HRDC’s prosthesis changed my life by giving me mobility and independence. Now, I inspire others who once saw disabilities with stigma, showing them that anything is possible.”*

A Boy’s Rebirth*: "Thanks to HRDC’s spine team, my congenital kyphoscoliosis was fixed, giving me a new lease on life. I am now confident, back in school, and determined to become a teacher.”*

A Young Woman’s Aspiration*: "HRDC corrected my leg deformity and helped me regain my dignity. Today, I aspire to become an advocate for change and help others like me.”*

A Mother’s Story (Clubfoot Case): *"HRDC’s affordable clubfoot care transformed my son’s life, allowing him to walk and run like any other child. It’s a beacon of hope for families like ours.”*

### 5.2 Testimonials from government officials

Honorable Gagan Thapa (Former Health Minister of Nepal): *“HRDC’s new Regional Rehabilitation Centre in Kohalpur is a testament to the fruitful partnership between HRDC and the government.”*

### 5.3 Testimonials from donors and partners

American Himalayan Foundation: *“We have been partnering with HRDC for over 35 years to enrich the lives of children with disabilities in Nepal. Their dedication to improving healthcare and empowering communities has profoundly impacted.”*

CBM: *“HRDC is a vital partner in providing high-quality care for children with physical disabilities, and we are proud of our long-standing collaboration for more than 30 years.”*

Miracle Feet: *“Since 2015, our partnership with HRDC has expanded access to quality clubfoot care for Nepalese children, ensuring impactful change.”*^6^

Local Government Contribution (Kohalpur & Itahari): *“HRDC’s decentralized services, supported by government-provided facilities, have enabled significant cost savings and improved accessibility.*”

## 6 Challenges and lessons learned

Over the past four decades, the efforts to provide inclusive and sustainable healthcare for children with disabilities, especially in resource-constrained areas like Nepal, have faced numerous challenges. Key focus areas have included financial, infrastructural, social, donor, policy, staffing, and skills-related issues. Addressing these challenges has been crucial to improving service delivery and ensuring long-term success. Throughout this journey, valuable lessons have been learned, offering essential insights for overcoming these obstacles.

**Table 1:** 

Challenges	Lessons learned
*Financial barriers*	*Partnership synergy*
High operational costs for specialized equipment	Private-nonprofit collaboration significantly reduces operational costs
Limited funding sources in resource-constrained settings	Diversified funding sources ensure long-term financial sustainability
Additional financial burden on families	Cost-sharing initiatives help reduce financial burdens on families and accommodate non-affording patients and families
Difficulty in maintaining financial sustainability	Shared resources and expertise maximize operational impact
*Infrastructural barriers*	*Infrastructure and access solutions*
Limited accessibility in remote areas of Nepal	Establish satellite clinics in key locations with consistent service quality (26, 27) and organize regular mobile health camps with a diverse team, including doctors, nurses, physiotherapists, prosthetists, CBR workers, and medical recorders.
Difficulty in maintaining consistent quality across satellite clinics	Telemedicine implementation ensures consistent service delivery
Challenges in coordinating central and satellite locations	Community-based rehabilitation networks improve service coordination
Transportation difficulties for patients	Development of robust referral and transport systems
*Social barriers*	*Community integration strategies*
Persistent stigma and discrimination against children with disabilities	Implement community-based rehabilitation (CBR) programs to promote social inclusion, complemented by awareness campaigns in schools, local communities, and other relevant platforms.
Cultural beliefs and superstitions affecting treatment acceptance	Cultural sensitivity in service delivery increases acceptance and trust
Limited awareness about treatments and rehabilitation options	Awareness campaigns and education initiatives address knowledge gaps
Social exclusion of children with disabilities	Schools and community organizations actively engage and integrate children
Educational barriers and limited integration opportunities	Dedicated child protection officers support integration and inclusion
*Donor challenges*	*Sustainable operations*
Inconsistent donor support	Diversified funding sources ensure financial resilience
Stringent donor reporting requirements	Clear accountability and reporting systems build trust with donors
Dependency on short-term funding commitments	Strategic planning ensures long-term donor engagement
*Policy challenges*	*Operational improvements*
Lack of comprehensive policies for disability care	Development of standardized protocols across facilities
Regulatory constraints in funding and operations	Advocacy and collaboration with policymakers strengthen support
Bureaucratic hurdles in project approvals	Streamlined administrative processes enhance efficiency
*Staffing challenges*	*Workforce development strategies*
Shortage of qualified healthcare professionals	Address this challenge through continuous training programs, scholarships, mentorship initiatives, fellowship opportunities, and fostering strong team bonding among staff to ensure long-term sustainability.
High turnover rate among healthcare workers	Implement targeted staff development programs to improve retention rates, including competitive compensation packages, opportunities for career advancement, regular professional development training, recognition and reward systems, flexible work arrangements, and fostering a supportive workplace culture.
*Skills gap*	*Continuous professional development*
Lack of specialized training opportunities	Regular capacity-building workshops ensure skill enhancement
Inadequate knowledge of advanced treatment protocols	Collaboration with international experts for skill transfer
Difficulty in adapting new technologies and methods	Ongoing technical training sessions ensure service quality adherence

## 7 Replicability of the model in other contexts and countries

To replicate the HRDC model in other regions, it is crucial to establish institutions adaptable to local contexts while ensuring compassionate care, strong leadership, and a commitment to maintaining high-quality services. The following strategies outline key components for building a sustainable system that can effectively address the needs of children with disabilities.

- *Establish Institutions and Build Local Capacity:*

- *Institutional Setup:* Establish a hospital or rehabilitation center focused on pediatric orthopedic care, providing a comprehensive range of services—including diagnostics, surgery, physiotherapy, and prosthetics—all under one roof. This integrated approach minimizes the need for patients to visit multiple locations, ensuring continuous care and reducing barriers to accessing services.
- *Capacity Building:* Develop a robust training infrastructure to cultivate local professionals in specialized disability care fields, such as orthopedic surgery, physiotherapy, and rehabilitation. Collaborating with national and international institutions can bring expertise, helping build a sustainable workforce that delivers high-quality care.
- *Compassionate Leadership:* Form a team dedicated to providing compassionate, patient-centered care while driving the institution’s mission. This leadership should prioritize inclusivity and accessibility for all children. Compassionate leadership ensures that staff remain motivated, fostering a holistic approach to patient care and enhancing the well-being of the children served.

- *Adaptation to Local Contexts:*

- *Cultural Sensitivity:* Tailor healthcare services to the local population’s cultural, social, and economic needs. Engage local communities to understand their specific challenges, such as stigma, and ensure that services are delivered in a culturally acceptable manner.
- *Community Engagement:* Work with community leaders and families to address the stigma and societal challenges surrounding disabilities. Community-based rehabilitation (CBR) workers can play a key role in early identification, follow-up, and awareness campaigns to reduce stigma and foster acceptance.
- *Focus on Quality Care:* Ensure that quality is always a top priority by establishing clear clinical guidelines, regular staff training, and continuous monitoring of outcomes. Providing high-quality care should remain the cornerstone of the institution’s operations, ensuring every child receives the attention they deserve.

- *Public-Private Partnerships:*

- *Collaboration with Private Institutions:* Partner with private hospitals and clinics to access advanced diagnostic services and medical equipment at subsidized costs. These partnerships will help reduce overhead costs and ensure the hospital remains financially sustainable.
- *Referral Systems:* Establish a referral system where children needing complex care can be referred from private institutions to non-profit hospitals, ensuring access to specialized treatment without financial barriers.

- *Comprehensive Care and Holistic Support:*

- *Medical and Rehabilitation Services:* Offer a wide range of services under one roof, including surgery, physiotherapy, occupational therapy, and prosthetics. Ensure that rehabilitation services focus on improving both physical and social functioning, helping children become active participants in their communities.
- *Social and Educational Support:* Beyond medical treatment, establish social rehabilitation programs, including a child protection policy to ensure children return to supportive home environments. Set up in-house educational programs for children during their stay and provide ongoing educational support to facilitate reintegration into schools.

- *Financial Sustainability and Cost-Effectiveness:*

- *Diversified Funding Sources:* Ensure long-term financial sustainability by securing funding through government support, private donations, grants, and international collaborations. Financial transparency and effective management are crucial to maintaining operations.
- *Cost Reduction Strategies:* Keep operational costs low by utilizing donated equipment, receiving subsidized medical supplies, and leveraging partnerships with private institutions to reduce overhead. Provide services at a low or no cost to patients, ensuring accessibility for those from low-income backgrounds.

- *Focus on Social Reintegration:*

- *Community Integration:* Provide ongoing support to children and families for successful reintegration into their communities. This includes ensuring access to mainstream education, vocational training, and participation in local activities.
- *Collaboration with Local Organizations:* Work closely with local disability organizations, schools, and government bodies to ensure that children with disabilities are welcomed and supported in their communities. Promote inclusive education and advocate for disability-friendly policies.

- *Leadership and Organizational Sustainability:*

- *Strong Vision and Leadership:* Ensure robust, compassionate leadership committed to the long-term vision of providing inclusive and quality care for children with disabilities. Leaders should inspire staff, engage with the community, and focus on patient-centered care.
- *Employee Development and Retention:* Invest in staff training and career development to ensure long-term success and low turnover. Encourage continuous professional growth and ensure that employees feel valued and motivated.
- *Quality Assurance:* Establish a rigorous system for monitoring the quality of care, patient outcomes, and employee performance. Continuous quality improvement should be embedded in the institution’s culture to maintain high standards of care.

- *Leverage Technology and Innovation:*

- *Telemedicine and Remote Support:* Use telemedicine to expand access to consultations and follow-up care for patients in remote areas. This ensures that children and families receive ongoing support without the burden of travel.
- *Data Management and Reporting Systems:* Implement electronic health records and a robust reporting system to track patient progress, service delivery, and financial management. Data-driven decision-making will improve patient outcomes and operational efficiency.
- *E-Learning Platforms:* Develop online platforms for training healthcare workers, caregivers, and families, providing them with up-to-date knowledge on disability care and rehabilitation.

- *Collaboration with Government and Advocacy:*

- *Policy Advocacy:* Advocate for policies that promote disability rights and access to inclusive healthcare. Work with local governments to integrate disability care into national healthcare strategies.
- *Partnerships with NGOs and INGOs:* Engage with international organizations to expand service reach, share resources, and influence public policy. Collaborate on funding, research, and advocacy efforts to improve the lives of children with disabilities.

By establishing institutions that offer compassionate, high-quality care and adapting to local contexts, the HRDC model can be successfully replicated in other countries. The emphasis on leadership, community engagement, financial sustainability, and a holistic approach to care will ensure long-term impact. By fostering partnerships with private institutions, leveraging technology, and advocating for inclusive policies, this model can help provide children with disabilities access to the care, support, and opportunities they deserve, leading to a more inclusive and compassionate society.

## 8 Discussion

The HRDC-B&B model represents an innovative approach to addressing healthcare barriers for children with physical disabilities in resource-limited settings. This discussion analyzes its effectiveness by comparing it with other healthcare delivery models and examining its unique contributions to addressing systemic barriers in pediatric disability care. The HRDC-B&B model achieves a 62% cost reduction compared to private healthcare institutions, significantly exceeding the cost-effectiveness of similar interventions.

The model’s superior cost-effectiveness can be attributed to its unique private-non-profit partnership structure. Unlike traditional models that rely heavily on either government funding or private payments, the HRDC-B&B model employs a diversified funding approach. This aligns with findings from the health system elements in Africa on sustainable healthcare financing in developing countries, which emphasizes the importance of multiple funding streams for long-term sustainability (28).

The model’s success in maintaining financial sustainability while providing affordable care demonstrates the viability of this approach. Studies by Newacheck et al. (29) indicate that families of children with disabilities spend 2–3 times more on healthcare compared to other families. The HRDC-B&B model’s ability to eliminate out-of-pocket expenses for underprivileged families while maintaining high-quality care addresses this critical barrier effectively. The HRDC-B&B model’s network of satellite clinics and mobile camps represents a more comprehensive approach to geographic accessibility than traditional centralized healthcare models. HRDC’s coverage extends to all 77 districts of Nepal through its rotating mobile clinic system.

Unlike standalone medical facilities, the model’s integration with community-based rehabilitation (CBR) workers creates a continuous care pathway. Research by Dambi and Jelsma (30) on healthcare delivery in Zimbabwe shows that such integrated approaches result in better treatment adherence than traditional hospital-based care models. The model’s use of telemedicine for connecting satellite clinics with the main center represents a modern approach to bridging geographic barriers. Studies indicate that telemedicine integration in rural healthcare offers numerous advantages over traditional in-person healthcare encounters for patients and providers. These include reduced travel costs and time, shorter waiting periods, minimized risk of communicable disease transmission, decreased overall time spent on consultations, and enhanced convenience (31), aligning with HRDC’s observed outcomes. The “under one roof” approach of HRDC differs significantly from the fragmented care models common in many LMICs (32, 33). Studies found that integrated care models result in better health outcomes for children with physical disabilities than fragmented care approaches (34). The model’s unique approach to professional staffing, where specialists work across both private and non-profit sectors, shows remarkably high retention rates. This contrasts with typical rural healthcare facilities in LMICs, which face high annual staff turnover rates (35). The model emphasizes community engagement and education beyond traditional medical models. Research on disability stigma in developing countries shows that community-integrated approaches can reduce social stigma (36). HRDC’s approach to cultural sensitivity and local integration aligns with the best practices identified in global health literature.

A study by Handtke et al. (37) found that culturally adapted healthcare models show better patient compliance than standard approaches. The model’s dual leadership structure and symbiotic relationship between private and non-profit sectors represent a unique approach to organizational sustainability; HRDC’s long-term sustainability demonstrates the effectiveness of this approach, which the study conducted by the Gray (38) also supports. The sharing of resources between private and non-profit entities in the HRDC-B&B model shows superior resource utilization compared to standalone facilities. Studies by Fattahi et al. (39) indicate that such resource-sharing models can achieve better resource utilization rates. The success of the HRDC-B&B model has significant implications for global health policy, particularly in developing countries. Its achievements in addressing multiple healthcare barriers simultaneously while maintaining financial sustainability suggest that similar models could be adapted for other contexts.

## 9 Conclusion

The HRDC-B&B model represents a significant advancement in addressing healthcare barriers for children with physical disabilities in resource-limited settings. Its comprehensive approach to addressing financial, geographic, and social barriers and its sustainable operational model provides valuable lessons for global health interventions. The model’s success in Nepal demonstrates that complex healthcare challenges can be effectively addressed through innovative partnerships and community-integrated approaches. The model’s achievements in cost reduction, geographic coverage, and quality of care exceed typical outcomes reported in global health literature, suggesting that its approach could serve as a template for similar interventions in other developing countries. However, successful replication would require careful adaptation to local contexts and strong commitment from both private and non-profit sectors.

## Data Availability

The original contributions presented in the study are included in the article/Supplementary material, further inquiries can be directed to the corresponding author.

## Footnotes

^1^English_Report_Wordfile (1).

^2^HRDC: Hospital & Rehabilitation Centre for Disabled Children (2024).

^3^B & B Hospital (2024).

^4^World of Children (2024), available at: https://worldofchildren.org/honoree/ashok-banskota/.

^5^World of Children (2015), available at: https://worldofchildren.org/2011-honoree-dr-ashok-banskota-receives-2014-star-impact-award/.

^6^Miraclefeet (2015), Nepal, available at: https://www.miraclefeet.org/countries/nepal.
