# Supplementary material for: Unlocking potential: innovative “private-non-profit” partnership for empowering children with disabilities in resource-limited settings in Nepal
Source: Front Public Health. 2025 Feb 19;13:1438992. doi: 10.3389/fpubh.2025.1438992 (PMC11879793; doi:10.3389/fpubh.2025.1438992)
Supplement: Supplementary file 1 [file Table_1.DOCX]

# HRDC Cost Efficiency and Its Impact

## Detailed Cost Analysis: HRDC vs. Private Institutions

### Assumptions and Definitions

***1. Private Institution Costs (Cₚ):***
 - Private healthcare includes several cost components:
 - Base Treatment Cost (B): The core cost of treatment, covering materials, labor, and overheads. This cost is common to both HRDC and private institutions.
 - Profit Margin (Pₚ): Private institutions add a profit margin of 20–25% to their total costs.
 - Doctor Charges (Dₚ): Additional fees paid to doctors, contributing 20–25% of total costs.
 - Asset Depreciation (Aₚ): Costs for maintaining and replacing equipment and infrastructure, contributing 10–12% of total costs.

 The total cost in private institutions can be expressed as:
 Cₚ = B + Pₚ + Dₚ + Aₚ

***2. HRDC Costs (Cₕ):*** - HRDC operates as a non-profit organization, supported by donor funding, which results in significant cost reductions:
 - No profit margin (Pₕ = 0): HRDC does not aim to generate profit.
 - Minimal doctor charges (Dₕ): HRDC employs salaried doctors or benefits from voluntary services, eliminating these costs.
 - No asset depreciation (Aₕ): HRDC’s infrastructure and equipment are funded through donations from NGOs, INGOs, and grants, removing this cost.

 Therefore, HRDC’s cost is limited to the base treatment cost:
 Cₕ = B

### *Step 1: Private Institution Costs*

For private institutions:
Cₚ = B + Pₚ + Dₚ + Aₚ

Substituting typical cost proportions:
 - Profit Margin (Pₚ) = k · Cₚ, where k = 0.20 to 0.25.
 - Doctor Charges (Dₚ) = m · Cₚ, where m = 0.20 to 0.25.
 - Asset Depreciation (Aₚ) = n · Cₚ, where n = 0.10 to 0.12.

Rearranging:
Cₚ = B / [1 - (k + m + n)]

Using maximum values (k = 0.25, m = 0.25, n = 0.12):
Cₚ = B / [1 - 0.62] = B / 0.38 ≈ 2.63B

### *Step 2: HRDC Costs*

At HRDC, since Pₕ = 0, Dₕ ≈ 0, and Aₕ = 0, the total cost simplifies to:
Cₕ = B

### *Step 3: Cost Reduction at HRDC*

The cost reduction percentage at HRDC is:
Cost Reduction (%) = [(Cₚ - Cₕ) / Cₚ] × 100

Substituting Cₚ = 2.63B and Cₕ = B:
Cost Reduction (%) = [(2.63B - B) / 2.63B] × 100

Simplifying:
Cost Reduction (%) = (1.63B / 2.63B) × 100 ≈ 62%

### *Step 4: The Role of Donor Contributions*

The remaining 38% of the cost not covered by HRDC’s operational reductions is funded through:
1. Donations from NGOs and INGOs: Covering asset depreciation, infrastructure, and equipment maintenance.
2. Grants: Secured from local and international organizations interested in scaling impactful programs.
3. Individual Contributions: Voluntary contributions from individuals who wish to support the initiative.
4. Patients’ Contributions: HRDC only accepts contributions from patients who are voluntarily willing to pay. No child is denied treatment due to an inability to pay.

This funding model ensures that HRDC:
- Provides care that is affordable and accessible.
- Offers treatment free of charge for underprivileged families.
- Creates an inclusive space for children with physical disabilities in Nepal.
